# Anticoagulation management and monitoring in ECMO: an international survey: communication from the ISTH SSC Subcommittee on Pediatric and Neonatal Thrombosis and Hemostasis

**DOI:** 10.1016/j.rpth.2026.103389

**Published:** 2026-02-17

**Authors:** Katherine Regling, Ronald Thomas, E. Vincent S. Faustino, Neil A. Goldenberg, Meera Chitlur

**Affiliations:** 1 Division of Hematology Oncology, Department of Pediatrics, Children’s Hospital of Michigan, Central Michigan University College of Medicine, Detroit, MI, United States; 2 Department of Pediatrics, Children’s Hospital of Michigan, Central Michigan University College of Medicine, Detroit, MI, United States; 3 Section of Critical Care Medicine, Department of Pediatrics, Yale School of Medicine, New Haven, CT, United States; 4 Johns Hopkins All Children's Institute for Clinical and Translational Research, St. Petersberg, FL and Departments of Pediatrics and Medicine, Johns Hopkins University School of Medicine, Baltimore, MD, United States

**Keywords:** anticoagulants, clinical laboratory techniques, ECMO, survey, therapeutics

## Abstract

**Objectives:**

The objective of this survey is to assess current practices in anticoagulation management, laboratory monitoring, and hematology involvement in extracorporeal membrane oxygenation (ECMO) patients.

**Methods:**

A cross-sectional international survey distributed between April 2023 to November 2023 to members of Hemostasis and Thrombosis Research Society, International Society on Thrombosis and Haemostasis, and Extracorporeal Life Support Organization. Respondents were pediatric and adult physicians involved in the care of ECMO patients.

**Results:**

Seventy-two survey responses were included with 83.3% of respondents being from an Extracorporeal Life Support Organization Institution. The respondents included both adult (41.7%) and pediatric (34.7%) physicians, as well as those who managed all age groups (23.6%). Approximately 53% reported including hematology as part of the care team. However, the majority reported that the hematologist is involved in complex cases only (72.2%). Respondents reported the routine utilization of an anticoagulation protocol (92%) and transfusion protocol (83%) to guide management decisions during ECMO therapy; however, management practices were variable. Both unfractionated heparin and bivalirudin were reported to be used routinely for anticoagulation at 92.2% and 52.9%, respectively.

**Conclusions:**

This survey demonstrated that hematology involvement for ECMO patients is still primarily for complicated cases. In addition, unfractionated heparin continued to be the anticoagulant of choice, with no significant changes in laboratory monitoring. Most centers utilize protocols for management, yet wide variability in ECMO management was again highlighted. Continued prospective and collaborative research efforts are needed to make advancements in treatment and outcomes, and to help streamline the care of this complex patient population.

## Essentials

• Management of ECMO is evolving with the use of bivalirudin.
• International survey of physicians to define current practices in pediatric and adult ECMO.
• Bivalirudin for use in ECMO was reported as being utilized by 53% of respondents.
• Hematology input for complex cases was 72%, but unable to determine if involvement has changed.

## 1 Introduction

Extracorporeal membrane oxygenation (ECMO) is a commonly utilized, life-saving therapy for critically ill adult and pediatric patients. Risks of bleeding and thrombosis are significant complications that cause morbidity and mortality [1,2]. Despite nearly a half century of ECMO experience, the monitoring and management practices surrounding anticoagulation during ECMO continues to vary greatly by institution [[3], [4], [5], [6]].

The most recent pediatric and adult surveys report that the vast majority of ECMO centers use unfractionated heparin (UFH) as the anticoagulant of choice [4,5]. However, the use of direct thrombin inhibitors, like bivalirudin, is increasing for a variety of reasons including lack of immunogenicity, reversible binding to thrombin that is independent of antithrombin, short half-life of 25 minutes (or less in neonates/infants) and primarily metabolism via non-organ proteolysis [7,8]. In addition to rapid changes in anticoagulation practice, there is increasing interest in hemostasis surrounding mechanical circulatory devices among hematology trained clinicians. Previously, the surveys by Bembea et al. [3] and Ozment et al. [5] reported that 18% to 27% of institutions involve the hematology/thrombosis team during ECMO only in complex cases.

Given the increasing use of direct thrombin inhibitors for anticoagulation in both adults and children, and general hematology interest, the primary aim of this survey was to characterize the current monitoring and treatment landscape in ECMO therapy.

## 2 Methods

### 2.1 Survey design and oversight

We conducted a cross-sectional international survey of physicians involved in the care of ECMO patients. The survey was exempt from the Institutional Review Boards at Central Michigan University (IRB No. 2023-008) and the Detroit Medical Center (IRB No. 20159). The survey questions were presented in different sections including institutional demographics (including questions surrounding the ECMO care team and patient population treated), the ECMO cannulation/circuit, anticoagulation management, and laboratory monitoring techniques. The questions were derived with the intent to determine utilization of newer anticoagulants, like bivalirudin, and the monitoring practices for these medications. We pilot tested the survey on 3 pediatric hematologists and revisions were completed prior to its final distribution.

### 2.2 Respondents and administration of the survey

The sample population included members of the Extracorporeal Life Support Organization (ELSO), Hemostasis and Thrombosis Research Society (HTRS), and International Society on Thrombosis and Haemostasis (ISTH). The survey was disseminated twice (approximately 3 months apart) by the respective societies, via the ELSO monthly newsletter, the HTRS email listserv, and the ISTH Scientific and Standardization Committees (SSC) for Pediatric and Neonatal Thrombosis and Hemostasis Subcommittee and Perioperative and Critical Care Thrombosis and Hemostasis Subcommittee MyISTH Community pages. The survey was distributed using web-based survey platform, Qualtrics^XM^, and was open from April 2023 to November 2023.

### 2.3 Statistical analysis

Survey response data were calculated and reported as ratios and proportions, given the categorical scale of the questions.

## 3 Results

**Figure 1:**
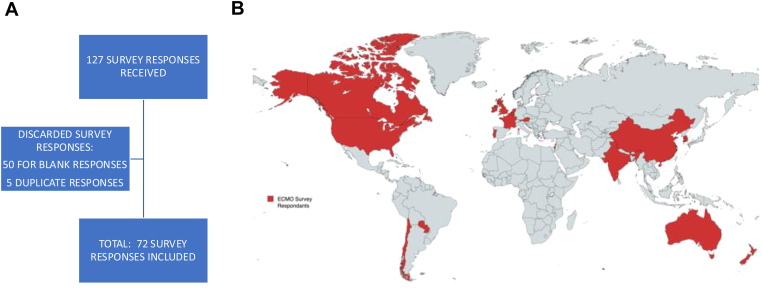
Survey response demographics.

This international survey included responses from 17 countries over 5 continents including North America (70%), Europe (12.8%), Asia (7.1%), Australia (7.1%), and South America (2.8%). A total of 72 survey responses were included (Figure 1) with 60/72 (83.3%) responders being from an ELSO Institution. The respondents included both adult (41.7%) and pediatric (34.7%) physicians, as well as those who managed all age groups (23.6%). The ECMO care team providers included physicians from multiple specialties (Table 1), with approximately 38/72 (52.8%) of institutions including hematology as part of the care team. When asked about the extent of hematology involvement, the majority of respondents reported that the hematologist is contacted in complex cases only (72.2%). Approximately 16.7% reported routine hematology involvement, while 20.8% reported that hematology is never contacted for management of ECMO/anticoagulation in these cases. Data for ECMO indications and ECMO circuit-related information are listed in Supplementary Table 1.

**Table 1:** The ECMO team, patient demographic, and cannulation practices.

Survey respondent demographics	Yes (%)
ELSO Institution (*N* = 72)	60 (83.3)
Access to ELSO database (*N* = 58)	40 (69)
Personally participate in ECMO management (*N* = 72)	72 (100)

ECMO team providers (*N* = 72)	Yes (%)
Hematology	38 (52.8)
PICU	38 (52.8)
NICU	18 (25)
Surgery	23 (31.9)
CV surgery	64 (88.9)
Other	28 (38.9)
Adult critical care^a^	15 (53.6)
Cardiology	6 (21.4)
Anesthesia	4 (14.3)
Transfusion medicine	2 (7.1)
Internal medicine	2 (7.1)
Pulmonology	1 (3.6)
Nephrology	1 (3.6)

Depth of hematology participation (*N* = 72)	Yes (%)
Never contacted	15 (20.8)
Complex cases only	52 (72.2)
Participates in regular rounds	5 (6.9)
Daily anticoagulation management	5 (6.9)
All anticoagulation decisions	2 (2.8)

Patient population treated (*N* = 72)	Yes (%)
Pediatrics	25 (34.7)
Neonates	0 (0)
Children	4 (5.6)
Neonates + children	21 (29.2)
Adults	30 (41.7)
All ages	17 (23.6)

Abbreviations: CPB, cardiopulmonary bypass; CV, cardiovascular; ECMO, extracorporeal membrane oxygenation; ECPR, extracorporeal cardiopulmonary resuscitation; ELSO, Extracorporeal Life Support Organization; NICU, neonatal intensive care unit; PICU, pediatric intensive care unit; VA, venoarterial; VAD, ventricular assist device; VAV, veno-arterio-venous; VV, venovenous; VVA, veno-veno-arterial.

a Includes critical care, cardiac and pulmonary intensivists.

### 3.1 Anticoagulation management

Approximately 92% (45/49 respondents) reported the routine use of an anticoagulation protocol to guide management decisions during ECMO therapy. The vast majority of centers reported routine use of UFH (92.2%, 47/51 respondents) and bivalirudin (52.9%, 27/51 respondents), in addition to low molecular weight heparin, argatroban, and fondaparinux as noted in Table 2. Reported indications for switching from UFH to bivalirudin included concern for heparin-induced thrombocytopenia (47%), heparin resistance (16%), and others (high risk of thrombosis, bleeding, low antithrombin, and specific clinical scenarios) (37%). The indications for use of UFH versus bivalirudin as a primary anticoagulant was not collected as a part of this survey. In addition, 79.6% (39/49 respondents) reported using ECMO therapy without the use of anticoagulation when there was a concern for or active severe bleeding present, in the trauma/post-operative setting, during severe thrombocytopenia, and other situations with increased risk of bleeding (Table 2).

**Table 2:** ECMO anticoagulation management.

Anticoagulation practices	Yes (%)
Anticoagulation protocol (*N* = 49)	45 (91.8)
Available anticoagulants (*N* = 51 for all subcategories)	
Unfractionated heparin	50 (98)
Low molecular weight heparin	35 (68.6)
Bivalirudin	41 (80.4)
Argatroban	27 (52.9)
Fondaparinux	13 (25.5)
Other	1 (2)
Nafamostat	1 (100)
Anticoagulants used routinely (*N =* 51 for all subcategories)	
Unfractionated heparin	47 (92.2)
Low molecular weight heparin	6 (11.8)
Bivalirudin	27 (52.9)
Argatroban	4 (7.8)
Fondaparinux	0 (0)
Other	1 (2)
Nafamostat	1 (100)
ECMO without anticoagulation (*N =* 49)	39 (79.6)
Unanswered	2 (5.1)
Active, severe bleeding	19 (48.7)
High-risk for bleeding	2 (5.1)
Post-operative	5 (12.8)
Trauma	2 (5.1)
Severe thrombocytopenia	1 (2.6)
VV Adult ECMO	2 (5.1)
Combination	6 (15.4)
Heparin starting dose (*N =* 43)	
5-10 units/kg/hr	6 (14)
10-15 units/kg/hr	14 (32.6)
15-20 units/kg/hr	3 (7)
20-25 units/kg/hr	5 (11.6)
>25 units/kg/hr	0 (0)
Combination/dependent	15 (34.9)
Bivalirudin starting dose^a^ (*N =* 35)	
0.05- 0.15 mg/kg/hr	13 (37.1)
0.15- 0.25 mg/kg/hr	14 (40)
0.25- 0.35 mg/kg/hr	6 (17.1)
0.35-0.45 mg/kg/hr	1 (2.9)
Other	1 (2.9)

Heparin: laboratory values monitored	Yes (%)
Activated clotting time (*N =* 48)	
Not used	30 (62.5)
Every 4-6 h	17 (35.4)
Every 8 h	0 (0)
Every 12 h	0 (0)
Daily	1 (2.1)
aPTT (*N =* 47)	
Not used	2 (4.3)
Every 4- 6 h	33 (70.2)
Every 8 h	1 (2.1)
Every 12 h	9 (19.1)
Daily	2 (4.3)
Anti-Factor Xa (*N =* 48)	
Not used	10 (20.8)
Every 4-6 h	24 (50)
Every 8 h	1 (2.1)
Every 12 h	6 (12.5)
Daily	7 (14.6)
TEG (*N =* 47)	
Not used	30 (63.8)
Every 4-6 h	1 (2.1)
Every 8 h	0 (0)
Every 12 h	0 (0)
Daily	16 (34)
TEG used to guide therapy (*N =* 48)	12 (25)
ROTEM (*N =* 47)	
Not used	38 (80.9)
Every 4- 6 h	1 (2.1)
Every 8 h	0 (0)
Every 12 h	0 (0)
Daily	8 (17)
ROTEM used to guide therapy (*N =* 48)	5 (10.4)
Bivalirudin: laboratory values monitored	Yes (%)
aPTT (*N =* 46)	
Not used	9 (19.6)
Every 4-6 h	35 (76.1)
Every 8 h	2 (4.3)
Every 12 h	0 (0)
Daily	0 (0)
DTT/DTI (*N =* 46)	
Not used	36 (78)
Every 4-6 h	5 (10.9)
Every 8 h	2 (4.3)
Every 12 h	1 (2.2)
Daily	2 (4.3)
TEG (*N =* 46)	
Not used	32 (69.6)
Every 4-6 h	0 (0)
Every 8 h	0 (0)
Every 12 h	1 (2.2)
Daily	13 (28.3)
TEG used to guide therapy (*N* = 46)	7 (15.2)
ROTEM (*N* = 46)	
Not used	42 (91.3)
Every 4-6 h	0 (0)
Every 8 h	0 (0)
Every 12 h	0 (0)
Daily	4 (8.7)
ROTEM used to guide therapy (*N* = 49)	4 (8.2)

Other laboratory parameters	Yes (%)
Complete blood count (*N* = 45)	
Not used	0 (0)
Every 4-6 h	10 (22.2)
Every 8 h	2 (4.4)
Every 12 h	18 (40)
Daily	9 (20)
Other	6 (13.3)
PT / INR (*N* = 45)	
Not used	0 (0)
Every 4-6 h	10 (22.2)
Every 8 h	2 (4.4)
Every 12 h	11 (24.4)
Daily	16 (35.6)
Other	6 (13.3)
Fibrinogen (*N* = 42)	
Not used	0 (0)
Every 4-6 h	7 (16.7)
Every 8 h	1 (2.4)
Every 12 h	11 (26.2)
Daily	18 (42.9)
Other	5 (11.9)
Antithrombin (*N* = 43)	29 (67.4)
Platelet function (*N* = 42)	9 (21.4)
Plasma free hemoglobin (*N* = 43)	34 (79.1)
CRP (*N* = 43)	18 (41.9)

aPTT, activated partial thromboplastin time; CRP, C-reactive protein; DTI, direct thrombin inhibitor time; DTT, dilute thrombin time; ECMO, extracorporeal membrane oxygenation; INR, international normalized ratio; PT, prothrombin time; ROTEM, thromboelastometry; TEG, thromboelastography; VV, venovenous.

a Based on normal renal function.

#### 3.2.1 UFH

Forty-three respondents (60%) commented on the starting doses used for UFH of which 34.9% (15/43) reported a combination of starting doses which was dependent upon the clinical scenario. However, significant differences in UFH starting dose were observed based on age. A lower starting dose of UFH at 5 to 20 units/kg/hr was utilized by 94.1% of centers that treat managed adults only, while only 35.7% of pediatric centers and 16.7% of centers that treat both adults and children reported using this dose range (*P* = .004). None of the adult centers reported starting doses >20 units/kg/hr, whereas, 14.3% of pediatric only centers and 25% of institutions that treat all age groups reported starting UFH doses of 21 to 25 units/kg/hr. In addition, variable starting doses based on clinical scenario were more likely to be used at pediatric only centers (46.7%) and institutions that treat both adult/pediatric patients (46.7%) compared to adult only institutions (6.7%).

#### 3.2.2 Bivalirudin

Thirty-five respondents (49%) commented on the starting doses used for bivalirudin (assuming normal renal function), with the majority starting between 0.15 and 0.25 mg/kg/hr (40%, 14/35 respondents). However, 37.1% (13/35 respondents) reported lower starting doses of 0.05 to 0.15 mg/kg/hr and 17.1% (6/35 respondents) reported higher starting doses of 0.25 to 0.35 mg/kg/hr (Table 2). One respondent reported starting doses between 0.125 and 0.25 mg/kg/hr and one reported starting doses of 0.35 to 0.45 mg/kg/hr. There were no significant variations identified in starting dose of bivalirudin based on age.

### 3.3 Laboratory parameters utilized for ECMO management

Details of the laboratory parameters utilized for management of ECMO are show in Table 2.

#### **3.3.1** General parameters

The frequency of assessment of general laboratory tests that are monitored during ECMO therapy including complete blood count, activated partial thromboplastin time (aPTT), prothrombin time, international normalized ratio (INR), anti-activated factor X (anti-FXa), and fibrinogen are shown in Table 2. Plasma free hemoglobin was assessed routinely in nearly 80% (34/43) of responding institutions. However, only 42% institutions routinely assessed C-reactive protein levels (18/43 respondents) and only 21% assessed for underlying platelet dysfunction (9/42 respondents) during ECMO therapy.

#### **3.3.2** Monitoring of UFH

**Figure 2:**
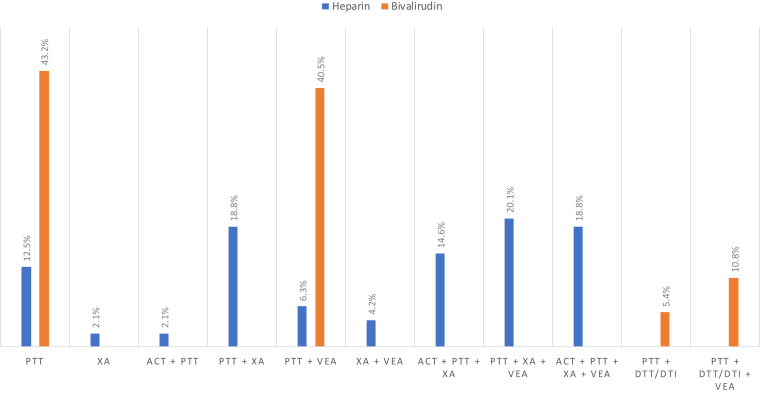
Various laboratory assay monitoring combinations for heparin and bivalirudin. ACT, activated clotting time; DTI, direct thrombin inhibitor time; DTT, dilute thrombin time; PTT, activated partial thromboplastin time; VEA, viscoelastic assay; XA, anti-factor Xa.

The laboratory parameter used most commonly for monitoring UFH was the aPTT at 95.7%, while the anti-FXa was utilized by 79.2%, and activated clotting time by 37.5%. The utilization of viscoelastic assays like thromboelastography (TEG) and thromboelastometry (ROTEM) were reported by 36.1% (17/47 respondents) and 19.1% (9/47 respondents) of centers, respectively. However, of the institutions that reported the routine use of viscoelastic assays, very few reported using these tests to help guide UFH anticoagulation therapy, TEG (25%, 12/48 respondents) and ROTEM (10.4%, 5/48 respondents). Only 7 respondents commented on assessment of specific parameters for the TEG including R-time (100%, 7/7 respondents), K-time (57.1%, 4/7 respondents), alpha angle (28.6%, 2/7 respondents), maximum amplitude (71.4%, 5/7 respondents), delta G (0%, 0/7 respondents), clotting index (14.3%, 1/7 respondents), and lysis 30 (14.3%, 1/7 respondents); with a majority (85.7%, 6/7 respondents) that used a combination of these parameters to help guide therapy. Of the 5 respondents who commented on using ROTEM to guide UFH therapy, the clotting time was the only parameter reported to be assessed. Various combinations of laboratory assay monitoring practices used can be seen in Figure 2.

#### **3.3.3** Monitoring of bivalirudin

The aPTT was the most common laboratory parameter used for monitoring bivalirudin (Figure 2) at 80.4%. Dilute thrombin time (DTT) (or direct thrombin inhibitor time [DTI]) was reported by 21.7%. Utilization of viscoelastic assays including TEG and ROTEM for monitoring bivalirudin was reported by 30.4% (14/46 respondents) and 8.7% (4/46 respondents) of centers, respectively. Of these centers, very few used TEG and ROTEM to help guide bivalirudin therapy, 15.2% (7/46 respondents) and 8.2% (4/49 respondents), respectively. Only 5 respondents commented on assessment of specific parameters for the TEG including R-time (100%, 5/5 respondents), K-time (80%, 4/5 respondents), alpha angle (20%, 1/5 respondents), maximum amplitude (60%, 3/5 respondents), delta G (20%, 1/5 respondents), clotting index (20%, 1/5 respondents), and lysis 30 (20%, 1/5 respondents); with all (100%, 5/5 respondents) that used a combination of these parameters to help guide therapy. Of the 4 respondents who commented on using ROTEM to guide bivalirudin therapy, the clotting time was the only parameter reported to be assessed.

### 3.4 Transfusion support

Overall, 82.6% (38/46 respondents) reported using a transfusion support protocol to guide when and with what product to replace deficiencies. Table 3 shows various transfusion thresholds and product replacement preferences. Interestingly, for both red blood cell transfusions (Pearson Chi Square = 0.073) and platelet transfusions (Pearson Chi Square = 0.244) the replacement thresholds were quite variable, and there were no significant differences between age groups treated (Supplementary Figure 1). Abnormal INR and/or fibrinogen levels were routinely replaced, with majority of institutions using either fresh frozen plasma or cryopreciptate.

**Table 3:** ECMO and transfusion support.

	Yes (%)
Transfusion support protocol (*N* = 46)	38 (82.6)
RBC transfusion thresholds (*N* = 44)	
Hb <6 gm/dL	0 (0)
Hb 6-7 gm/dL	7 (15.9)
Hb 7-8 gm/dL	14 (31.8)
Hb 8-10 gm/dL	13 (29.5)
Other	10 (22.7)
Platelet transfusion thresholds (*N* = 44)	
PC <10 k	2 (4.5)
PC 10-20 k	4 (9.1)
PC 20-50 k	10 (22.7)
PC 50-75 k	8 (18.2)
PC 75-100 k	11 (25)
Other	9 (20.5)
Plasma / cryoprecipitate to correct inr (*N* = 44)	35 (79.5)
Plasma to correct INR (*N* = 26)	
INR 1-1.5	1 (3.8)
INR 1.5-2	7 (26.9)
INR 2-2.5	12 (46.2)
INR 2.5-3	3 (11.5)
INR >3	3 (11.5)
Cryoprecipitate to correct INR (*N* = 11)	
INR 1-1.5	0 (0)
INR 1.5-2	2 (18.2)
INR 2-2.5	4 (36.4)
INR 2.5-3	1 (9.1)
INR >3	4 (36.4)
Fibrinogen desired range (*N* = 42)	
50-100 mg/dL	1 (2.4)
100-150 mg/dL	13 (31)
150-200 mg/dL	13 (31)
>200 mg/dL	9 (21.4)
Other	6 (14.3)
Products for fibrinogen replacement (*N* = 42 for all subcategories)	
FFP	11 (26.2)
Cryoprecipitate	35 (83.3)
Fibrinogen concentrates	11 (26.2)
Other	1 (2.4)
Antithrombin replacement (*N* = 44)	
Always	7 (15.9)
Heparin resistance	23 (52.3)
Thrombosis	1 (2.3)
Do not replace	13 (29.5)
Antithrombin products utilized (*N* = 40)	
AT concentrates	27 (67.5)
FFP	2 (5)
Combination	5 (12.5)
Not replaced	6 (15)
Heparin dose is adjusted if AT is replaced (*N* = 42)	11 (26.2)

AT, antithrombin III; Hb, hemoglobin; INR, international normalized ratio; PC, platelet count; RBC, red blood cells.

Of 43 respondents, 67% reported routine monitoring of antithrombin levels. Despite this, antithrombin replacement was institution dependent. Of the centers that routinely monitored antithrombin levels, 24.1% always replaced, 62.1% replaced only for concerns of heparin resistance, and 0% for thrombosis. Comparatively, antithrombin replacement at centers that did not routinely monitor levels, 0% always replaced, 35.7% replaced only for concerns of heparin resistance, and 7.1% replaced for thrombosis. As would be expected, antithrombin replacement was significantly higher (*P* = .005) at institutions where antithrombin levels were routinely monitored compared to institutions that did not monitor antithrombin levels at 86.2% versus 42.9%, respectively. In those institutions where antithrombin replacement was utilized, the majority used antithrombin concentrates (67.5%). After replacement, only 26.2% of respondents reported adjustment of heparin dosing.

## 4 Discussion

This survey was developed to describe the real world utilization and monitoring of medications and the teams involved in anticoagulation management in ECMO cases. The PEACE Collaborative has published extensively in the field of monitoring and management of ECMO in children and neonates. Their consensus reports have developed both circuit technology, anticoagulation, and general management guidelines [[9], [10], [11]]. These efforts have been consensus recommendations for practice but do not necessarily reflect real world practice. As hematologists, we were interested in understanding the level of involvement of hematology in ECMO management. Routine involvement by hematology remained very low. Our survey showed hematology help with daily decision making in 6.9% (5/72 respondents), participation in daily rounds in 6.9% (5/72 respondents), all decision making in 2.8% (2/72 respondents), and never contacted in 20.8% (15/72 respondents). These findings are similar to the previously published survey by Bembea et al. that reported hematology help with daily decision making in 13.6% (16/118 respondents), participation in daily rounds in 3.4% (4/118 respondents), all decision making in 1% (1/118 respondents), and never contacted in 4.2% (5/118 respondents) [3]. However, when looking at hematology involvement for complex cases only, our survey reported involvement in 72%, whereas, Ozment et al. [5] and Bembea et al. [3] previously reported hematology involvement in 18 to 27%. This survey was not designed to determine any differences between programs where hematology played a major role and those which did not, and given the low response rate, no conclusions can be made on if hematology involvement is truly increasing. In addition, further statistical analysis of these data was not done due to differences in population surveyed and possible differences in how questions were asked. It is unknown if the level of hematology involvement is impacted by the use of various institutional guidelines but would be a perspective to consider in future assessments.

In regard to anticoagulant use for ECMO, UFH was still the most widely used anticoagulant. However, approximately 80% of respondents reported access to bivalirudin and 53% of respondents reported routine use of bivalirudin with variable targets and indications for consideration of alternative anticoagulants to UFH. The primary indications for transitioning from UFH to bivalirudin included concern for heparin induced thrombocytopenia and heparin resistance. However, specific indications for use of UFH versus bivalirudin as a primary anticoagulant was not collected as a part of this survey and would be important to assess in future studies.This may be a reflection of the insufficient data to make specific recommendations for anticoagulant recommendations, as noted by the PEACE group [10]. Continued investigation surrounding bolus and maintenance dosing and safety and efficacy of direct thrombin inhibitors in comparison to UFH, would help to streamline anticoagulant choice and their use in our practices.

On the laboratory side, the survey reiterated that the aPTT (95.7%) and anti-FXa (79.2%) remained the most commonly used assays for monitoring of heparin, either alone or in combination with other assays. However, our survey found that the monitoring of UFH using the ACT has decreased compared to previous surveys, which is likely related to the many testing limitations associated with the ACT [[12], [13], [14], [15]]. Interestingly, the routine replacement of antithrombin to potentiate the heparin effect has become more controversial in the last decade. However, approximately two-thirds of institutions reported regular monitoring of antithrombin levels, yet, only 24% of these report routine replacement with antithrombin. This may be due to concern for increased bleeding risk without improvement in overall outcomes. Continued prospective studies on antithrombin replacement, incidence of complications and effects on overall outcomes would add benefit and help to standardize treatment protocols. Similar to UFH, the aPTT is the primary monitoring assay for bivalirudin, which may be due to the lack of accessibility to run more specific testing assays like the DTT/DTI [16]. Improved access to specialized coagulation laboratories and advanced training of medical technologists to run these specific assays may allow for shorter time to therapeutic anticoagulation, reduced dosing adjustments and possibly improved overall outcomes. Our survey showed that other viscoelastic assays, like TEG and ROTEM are being assessed in up to a third of institutions, however, their influence on treatment decisions remained minimal and without standardized guidelines. Continued prospective investigation of how these viscoelastic assays may benefit day-to-day management is imperative.

Interestingly, this study found that the majority of institutions have developed and utilize transfusion support guidelines, however, the replacement thresholds showed significant variation and age did not appear to have an impact. Data from ongoing studies surrounding platelet and red blood cell transfusion support thresholds will be important on future replacement in both adult and pediatric populations.

### 4.1 Limitations

There are several limitations to this study. First, the overall response rate was low, given the number of members that participate in the societies where the survey was distributed. This study was distributed through ELSO, HTRS, and ISTH in hopes to capture both adult and pediatric treating physicians as well as input from hematologists who assist in the care of ECMO patients worldwide. However, this may falsely have increased the role that hematologists are currently playing in the management of these patients and limited the number of non-hematologists who manage ECMO outside of ELSO registered ECMO centers. In addition, the survey pre-testing was completed by pediatric hematologists. Also, most survey responses were identifiable, however, we can not rule out the possibility of some unidentifiable duplicates. Lastly, there were several survey questions with missing data which may have affected reported similarities and/or changes in management of these complex patients. Like most surveys, the data may reflect stated practice and not actual practice.

## 5 Conclusions

ECMO is a complex therapeutic modality with significant variability in circuit technology and anticoagulation monitoring practices. Routine hematology involvement remained low, and given the low response rate, no conclusion can be made if the involvement has increased compared to previous surveys. Increased involvement from hematology, even in “routine” cases, may allow for increased understanding of the nuances of ECMO management and its complications from the hematology team perspective. With routine involvement, hematology may be able to add value in regard to anticoagulation dosing/monitoring and the use of transfusions and may potentially better serve complex cases in the future. This international survey demonstrated that the most common anticoagulant used is UFH and monitoring UFH with aPTT and anti-FXa remain the standard at most institutions. However, routine use of bivalirudin was reported at 53% and may be an indication that a similar survey in the next 5 years may be valuable to determine a change in practice. Continued prospective and collaborative research efforts are needed to make advancements in treatment and outcomes, as well as, to help streamline the management and monitoring of this complex patient population.
