# Supplementary material for: Anticoagulation management and monitoring in ECMO: an international survey: communication from the ISTH SSC Subcommittee on Pediatric and Neonatal Thrombosis and Hemostasis
Source: Res Pract Thromb Haemost. 2026 Feb 17;10(2):103389. doi: 10.1016/j.rpth.2026.103389 (PMC12993287; doi:10.1016/j.rpth.2026.103389)
Supplement: Supplementary Figures [file mmc1.pdf]

Supplemental Figure 1. Age-based RBC and Platelet Transfusion Thresholds

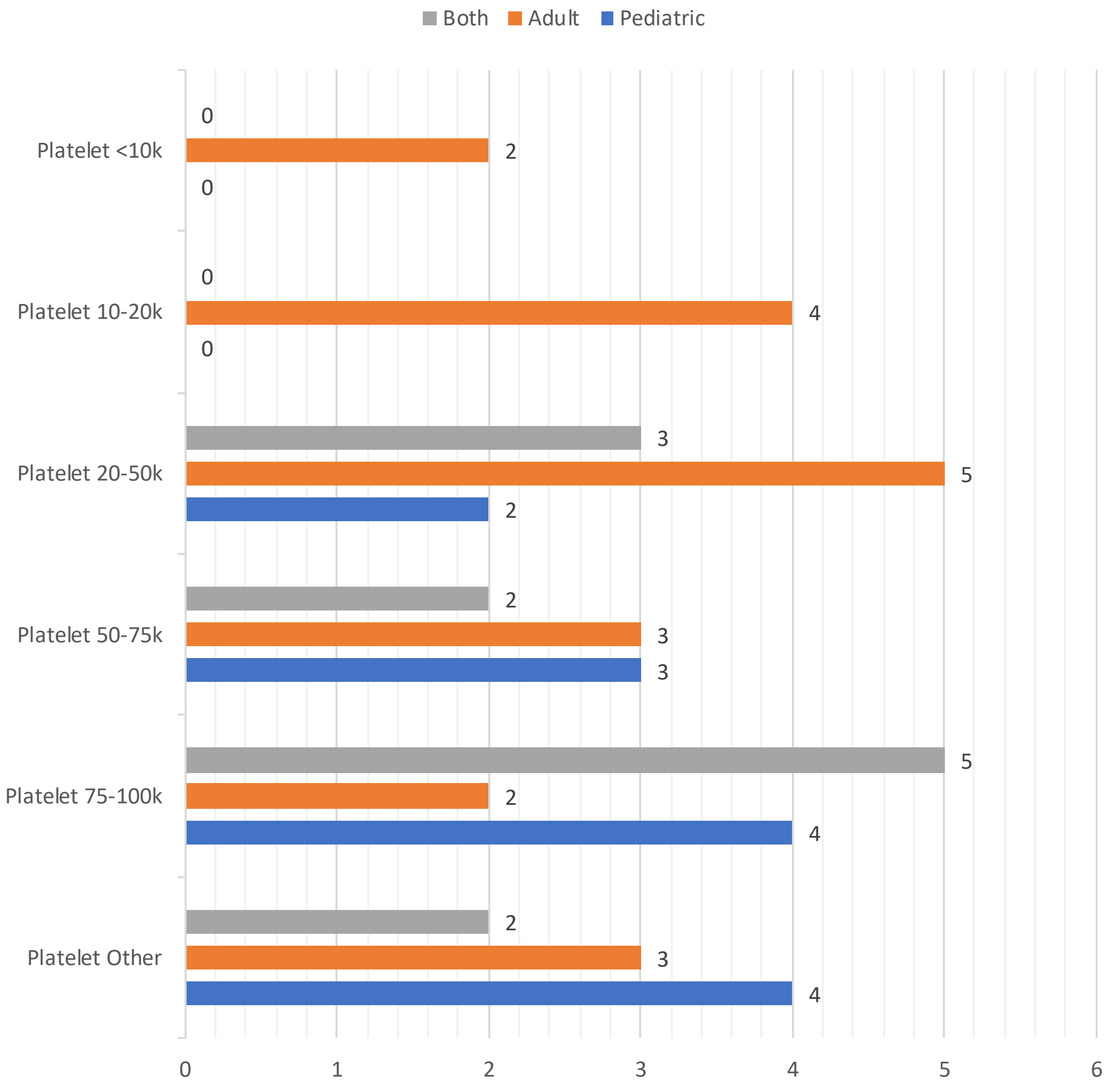

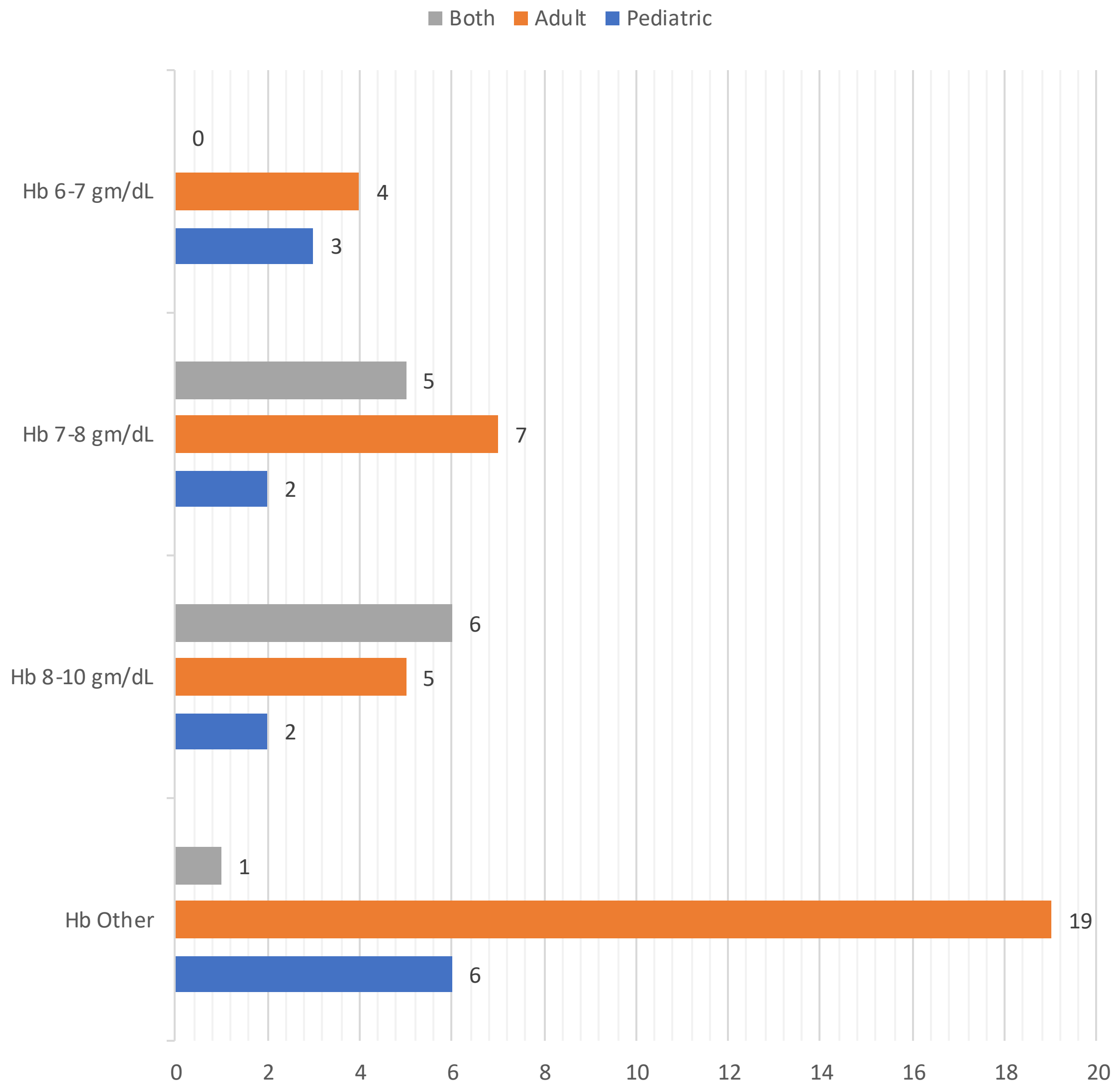

Abbreviations: Hb, hemoglobin
